# Human gut microbiota-reactive DP8α regulatory T cells, signature and related emerging functions

**DOI:** 10.3389/fimmu.2022.1026994

**Published:** 2022-11-21

**Authors:** Francine Jotereau, Joudy Alameddine, Raluca Teusan, Annabelle Pédron, Nicolas Jouand, Frédéric Altare, Emmanuelle Godefroy

**Affiliations:** ^1^ Nantes Université, Univ Angers, INSERM, CNRS, Immunology and New Concepts in ImmunoTherapy, INCIT, UMR 1302/EMR6001, Nantes, France; ^2^ Nantes Université, CHU Nantes, INSERM, CNRS, SFR Santé, Inserm UMS 016, CNRS UMS 3556, Nantes, France; ^3^ Cytocell, BioCore, Nantes Université UMS 3556, Inserm US016, CNRS UAR 3556, CHU Nantes, SFR Santé François BONAMY, Nantes, France

**Keywords:** human gut microbiota-specific Tregs, Faecalibacterium prausnitzii, gut microbiota, cytotoxic CD4^+^ T cells, IgA, IBD

## Abstract

In mice, microbiota-induced Tregs both maintain intestinal homeostasis and provide resistance to immuno-pathologies in the adult. Identifying their human functional counterpart therefore represents an important goal. We discovered, in the human colonic lamina propria and blood, a FoxP3-negative IL-10-secreting Treg subset, which co-expresses CD4 and CD8α (hence named DP8α) and displays a TCR-reactivity against *Faecalibacterium prausnitzii*, indicating a role for this symbiotic bacterium in their induction. Moreover, supporting their role in intestinal homeostasis, we previously reported both their drastic decrease in IBD patients and their protective role *in vivo* against intestinal inflammation, in mice. Here, we aimed at identifying the genomic, phenotypic and functional signatures of these microbiota-induced Tregs, towards delineating their physiological role(s) and clinical potential. Human *F. prausnitzii*-reactive DP8α Treg clones were derived from both the colonic lamina propria and blood. RNA-sequencing, flow cytometry and functional assays were performed to characterize their response upon activation and compare them to donor- and tissue-matched FoxP3^+^ Treg clones. DP8α Tregs exhibited a unique mixed Tr1-like/cytotoxic CD4^+^ T cell-profile and shared the RORγt and MAF master genes with mouse gut microbiota-induced FoxP3^+^ Tregs. We revealed their potent cytotoxic, chemotactic and IgA-promoting abilities, which were confirmed using *in vitro* assays. Therefore, besides their induction by a *Clostridium* bacterium, DP8α Tregs also partake master genes with mouse microbiota-induced Tregs. The present identification of their complete signature and novel functional properties, should be key in delineating the *in vivo* roles and therapeutic applications of these unique human microbiota-induced Tregs through their study in pathological contexts, particularly in inflammatory bowel diseases.

## Graphical Abstract

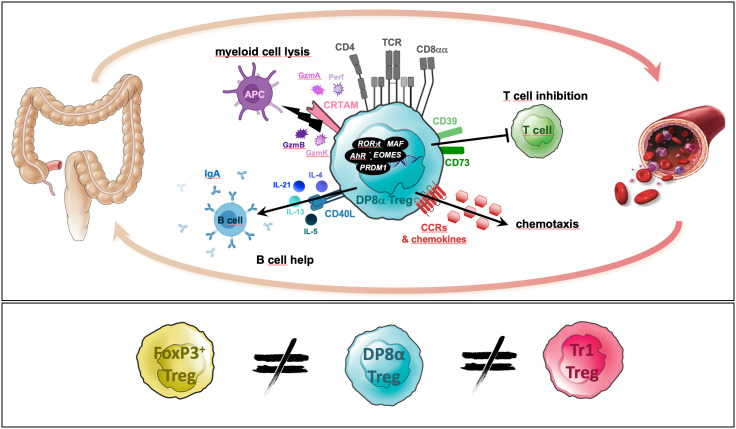

## Introduction

Observational studies unveiled strong associations between gut microbiota dysbiosis, including decreased *F. prausnitzii* levels, and common metabolic and chronic inflammatory disorders, suggesting that intestinal microbiota composition affects human health. Although, mechanistic understanding of these associations remains limited, one mechanism, identified in mice, relies on microbiota-induced immune imprinting at weaning, which protects against inflammatory diseases later in life (1). Such imprinting was shown to partly depend on the induction, by *Clostridium* IV and XIVa members, of mouse RORγt-expressing FoxP3^+^ Tregs, which prevent colitis and limit Th2-mediated responses such as allergic diseases (1, 2). Strikingly, we have identified, in the human colonic mucosa and blood, a FoxP3-negative IL-10-secreting Treg subset, named DP8α based on its co-expression of CD4 and CD8α, sharing major properties with mouse gut RORγt^+^ Tregs. Indeed, DP8α Tregs are abundant in the colonic lamina propria and are induced by a *Clostridium* IV member, *Faecalibacterium prausnitzii*, as indicated by their biased TCR reactivity against this bacterium (3), together with the ability of this bacterium to induce IL-10- and IL-27-secreting tolerogenic dendritic cells (4). Moreover, suggesting their role in IBD prevention, we described a specific drastic reduction of DP8α Tregs in both colon and blood of IBD patients (3, 5, 6) and showed that their transfer protected mice against experimental colitis (7). Therefore, ascertaining the physiological roles of these unique human microbiota-induced Tregs appears critical.

To this end, we performed here an in-depth characterization of DP8α Tregs to delineate their regulatory potential through genome-wide transcriptomic, phenotypic and *in vitro* functional studies, using clones isolated from both healthy colonic lamina propria and blood, as compared to donor-matched classical FoxP3^+^ Treg clones.

## Methods

### Colon sample collection and processing

Normal colonic mucosa was surgically resected from six colorectal cancer patients at approximately 10cm from the tumor. Samples were part of a tissue biocollection registered by the French Ministry for Higher Education and Research (DC-2014-2206), with approval from the ethic committee (CPP Ouest IV-Nantes) and the institutional board of Nantes University Hospital. Tissues were processed in accordance with Helsinki declaration. Each patient signed an informed consent form. The lamina propria was separated from the epithelium after incubation in 1mM EDTA PBS buffer (20min), minced into fragments and washed with RPMI containing 10μg/ml penicillin and 0.1mg/ml gentamycin (Sigma-Aldrich). Lamina propria fragments were digested with collagenase IV (1mg/ml; Sigma-Aldrich), with shaking at 37°C. Mucus and debris were removed by filtration through a 40μm-cell strainer (BD). Viable lamina propria-derived lymphocytes (LPLs) were obtained through Ficoll gradient centrifugation.

### Production of human Treg clones

To isolate *F. prausnitzii*-reactive DP8α Treg clones from blood, purified VPD-stained CD4^+^ T cells, comprising DP8α Tregs, were co-cultured with purified autologous CD14^+^ monocytes loaded overnight with *F. prausnitzii* (1:1 ratio). Five days later, VPD^LOW^ CD3^+^/CD4^+^/CD8α^LOW^ cells were cloned from four healthy donors using the Aria III cell sorter (**Figure S1**). Alongside, from the same donors, CD3^+^/CD4^+^/CD8^-^/CD25^HIGH^/CD127^LOW^ classical FoxP3^+^ Tregs were similarly cloned (**Figure S1**). DP8α Tregs and FoxP3^+^ Tregs were also cloned from freshly-dissociated and sorted colonic LPLs (**Figure S1**). Clones were then amplified on feeder cells (6). After 4 weeks, resting clones were screened for their response to autologous monocytes presenting *F. prausnitzii* and their FoxP3 expression.

### Reagents

Cells were cultured in RPMI-1640 supplemented with 5% human serum, 2mM L-glutamine and 10μg/ml penicillin-streptomycin (Gibco). rhIL-2 was used for T cell culture and expansion. Violet Proliferation Dye 450 (VPD) (1μM, BD Bioscience), Brefeldin A (10μg/ml, Sigma-Aldrich) and 4% paraformaldehyde (Sigma-Aldrich) were used. POM-1 and AB-680 (MedChemExpress) drugs, which inhibit CD39 and CD73, respectively, were used as indicated in the figure legends.

### Antibodies

For surface staining, cells were washed and stained for 40min at 4°C in PBS/0.1% BSA with the following antibodies: anti-CD3 (clone UCHT1, Becton Dickinson), anti-CD4 (clone 13B8.2, Beckman Coulter), anti-CD8α (clone RPA-T8, Becton Dickinson), anti-CCR6 (clone G034E3, Biolegend), anti-CXCR6 (clone K041E5, Biolegend), anti-CXCR5 (clone 51505, R&D Systems), anti-CD39 (clone A1, Biolegend), anti-CD73 (clone AD2, Biolegend), anti-CD27 (clone O323, Biolegend), anti-CD40L (clone 24-31, Abcam), anti-CD19 (clone SJ25C1, Biolegend), anti-CD38 (clone HB-7, Biolegend), anti-CD25 (clone BC96, eBiosience), anti-CD127 (clone HIL-7R-M21, Becton Dickinson), anti-CRTAM (clone 210213, R&D Systems), and anti-CCR5/CD195 (clone 3A9, Becton Dickinson).

For intracellular staining, cells were fixed in 4% paraformaldehyde for 10min, washed and stained for 40min at RT in PBS/0.1% BSA/0.1% saponin with anti-IFNγ-APC (clone B27, Becton Dickinson), anti-CCL4/MIP-1β (clone D21-1351, Becton Dickinson), anti-CCL5/RANTES (clone 2D5, Becton Dickinson), anti-Granzyme A (clone CB9, Biolegend), anti-Granzyme B (clone GB11, Becton Dickinson), anti-Granzyme K (clone GM26E7, Biolegend), anti-IL-2 (clone MQ1-17H12, Becton Dickinson), anti-IL-4 (clone 8D4-8, Biolegend), anti-IL-5 (clone TRFK5, Biolegend), anti-IL-13 (clone JES10-5A2, Biolegend), anti-IL-21 (clone eBio3A3-N2, eBiosience), and anti-Perforin (clone δG9, Becton Dickinson). For transcription factors, cells were fixed and permeabilized using the Foxp3/Transcription Factor Buffer Set (eBioscience) and the following antibodies: anti-FoxP3 (clone 259D/C7, Becton Dickinson), anti-RORγt (clone AFKJS-9, eBioscience), anti-Eomes (clone WD1928, eBioscience) and anti-ID2 (clone ILCID2, eBioscience).

Fluorescence was measured on a FACS LSR II flow cytometer and analyzed using the FlowJo and Diva softwares (Becton Dickinson). Relative fluorescence intensity (RFI) corresponds to the mean fluorescence intensity (MFI) obtained with the specific antibody divided by the MFI obtained with the isotypic control.

### ELISAs

T cells were stimulated using coated anti-CD3 (clone OKT3, 1μg/ml, eBioscience) for 24-48h at 37°C. Supernatants were tested for their IL-10 and IFNγ contents using the Ready-Set-Go ELISAs according to the manufacturer’s guidelines (eBioscience). XCL1, total hIgA and hIgG were measured using ELISA kits (Invitrogen) according to the manufacturer’s instructions.

### RNA extraction

Clones were stimulated or not with 0.5μM OKT3 for 4h, washed extensively and dry pellets were frozen at -80°C. mRNA was extracted from all samples at once (to avoid batch effects) using the NucleoSpin RNA kit (Macherey-Nagel) and quantified with a NanoDrop.

### Inhibition of T cell proliferation

Freshly sorted CD4^+^ T cells (Miltenyi) were stained with 1μM VPD before being co-cultured with Treg clones with 20UI/ml rhIL-2, with or without CD3/CD28 microbeads (Miltenyi). In some cases, CD39 or CD73 inhibitors were added. VPD dilution was assessed 5 days later to determine proliferation.

### Statistical analyses

Statistical analyses were performed using GraphPad Prism version 9.3.0. Most single comparisons were performed using 2-sided t-tests or Mann-Whitney tests, as indicated in figure legends. For multiple comparisons, one-way ANOVA (either Friedman tests for matched data or Kruskal-Wallis tests when there are no matching or pairing), followed by Dunn’s multiple comparison tests to obtained adjusted p values were used, as indicated in figure legends. p<.05 was considered statistically significant.

### 3’seq RNA Profiling

3’seq-RNA Profiling protocol was performed as described (8). Libraries were prepared from 10ng total RNA in 4µl. The mRNA poly(A) tails were tagged with universal adapters, well-specific barcodes and unique molecular identifiers (UMIs) during template-switching reverse transcriptase. Barcoded cDNAs from multiple samples were pooled, amplified and tagmented using a transposon-fragmentation approach, which enriches for cDNA 3′ ends. 100ng of full-length cDNA were used as input to the Nextera DNA Sample Prep kit (Illumina), which enriches for cDNA 3′ ends. Library’s size was controlled on 2200 Tape Station System (Agilent Technologies). A library of 350–800bp length was run on an Illumina HiSeq 2500, using a Hiseq Rapid SBS Kit v2-50 cycles and a Hiseq Rapid PE Cluster Kit v2 according to the manufacturer’s protocol (Denaturing and Diluting Libraries for the HiSeq^®^ and GAIIx, Part # 15050107 v03 protocol, Illumina).

### Bioinformatics

Raw fastq pairs match the following criteria: the 16 bases of the first read correspond to six bases for a designed sample-specific barcode and 10 bases for a unique molecular identifier (UMI). The second read (58 bases) corresponds to the captured poly(A) RNA sequence. Bioinformatics steps are performed using a snakemake pipeline (https://bio.tools/3SRP). We performed demultiplexing of these fastq pairs to generate one single-end fastq for each sample. These fastq files were then aligned with bwa to the reference mRNA refseq sequences and the mitochondrial genomic sequence, both available from the UCSC download site.

Gene expression profiles were generated by parsing the alignment files (bam) and counting, for each sample, the number of UMIs associated with each gene. Reads aligned on multiple genes, containing more than three mismatches with the reference sequence or having a polyA pattern were discarded. Finally, a matrix containing the counts of all genes on all samples was produced. The expression values, corresponding to the absolute abundance of mRNAs in all samples, were then ready for further gene expression analysis.

The R package DESeq2 (9) was then used for the differential analyses.

Data are available at GSE197906.

### Cytotoxic assays

T cell clones were co-cultured with 100,000 indicated target cells at indicated ratios at 37°C, with or without CD3/CD28 activation (T-Cell TransAct polymeric nanomatrix inducing low to no death of activated T cells). Supernatants were harvested 18h later and cell death was measured using a bioluminescent, non-destructive cytolysis assay kit designed to measure the release of the adenylate kinase from damaged cells (ToxiLight™ BioAssay Kit, Lonza). The ToxiLight Lysis Control Set (Lonza) was used to determine 100% lysis. Lysis percentages were calculated as follows:

*((Luminescence read - Luminescence of target cells alone lysed spontaneously x 100)/(Luminescence of totally lysed target cells – (Luminescence of target cells alone lysed spontaneously + Luminescence of corresponding T cells alone lysed spontaneously)*

### T/B co-culture assays

Freshly sorted CD3^-^/CD19^+^/CD27^-^ naïve B cells (20x10^3^ cells/well) were incubated at a 1:1 ratio with autologous sorted DP8α Tregs (CD3^+^/CD4^+^/CD8α^LOW^/CCR6^+^/CXCR6^+^), Tfh cells (CD3^+^/CD4^+^/CD8^-^/CXCR5^+^) or FoxP3^+^ Treg clones in RPMI-1640 supplemented with 10% fetal bovine serum (to avoid any contamination with human Ig) in the presence of an agonistic anti-BCR (anti-IgA/IgG/IgM) antibody (Jackson ImmunoResearch), with or without CD3/CD28-stimulation. At day 7, frequency of CD3^-^/CD19^INT^/CD38^+^ plasmablasts among total CD19^+^ B cells was measured, and total IgG and IgA were measured in the supernatants.

## Results

### Treg clone selection and characterization

We selected 6 DP8α and 4 FoxP3^+^ Treg clones derived from colonic lamina propria lymphocytes (LPLs) from five different donors, and matched-pairs of DP8α and FoxP3^+^ Treg clones from the blood from four healthy donors, as described in the Methods section.

**Figure 1:**
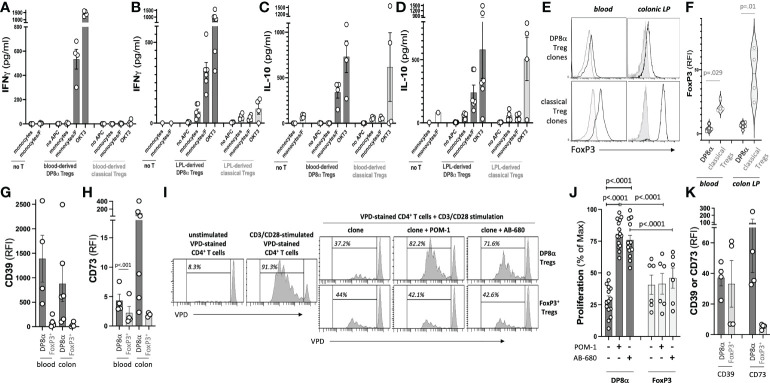
Treg clone selection and characterization. The gating strategy to sort *F. prausnitzii*-reactive DP8α and classical FoxP3^+^ Tregs used for clone production is described in the Methods’ section and shown in **Figure S1**. **(A-D)** Clones were screened by ELISA for their IFNγ **(A, B)** and IL-10 **(C**, **D)** responses to *F. prausnitzii* or anti-CD3 stimulation (clone OKT3). **(E**, **F)** FoxP3 expression was assessed by intracellular staining. Representative histogram plots examples (left) and all data (right) are shown **(G, H)**. CD39 **(G)** and CD73 **(H)** expression by clones measured using surface immunostaining. **(I**, **J)**. CD3/CD28-stimulated VPD-stained CD4^+^ T cells were co-cultured with Treg clones (ratio 1:1) in the presence or not of CD39 and CD73 inhibitors, namely POM-1 (30μM) and AB-680 (100nM), respectively, as indicated. Proliferation was evaluated by VPD dilution. Representative histogram plots examples **(I)** and all tested conditions (i.e., 5 DP8α clones and 2 FoxP3^+^ clones each co-cultured with CD4^+^ cells from 3 distinct donors) **(J)** are shown. **(K)** CD39 and CD73 expression by gated CD3^+^/CD4^+^/CD8α^LOW^/CCR6^+^/CXCR6^+^ DP8α and CD3^+^/CD4^+^/CD25^HIGH^/CD127^LOW^ classical Tregs among *ex vivo* PBMCs is shown. RFI corresponds to the ratio: MFI obtained with the relevant antibody over MFI for the corresponding isotype control. Mann-Whitney tests were used for single comparisons, while one-way ANOVA followed by Dunn’s multiple comparison tests were performed for 1J, p<.05 was considered statistically significant.

All DP8α clones responded to *F. prausnitzii* (**Figures 1A-D**), and lacked FoxP3 expression (mean RFI=4.46 ± 1.27 and 6.31 ± 0.95 for blood- and LPL-derived clones, respectively), as compared to FoxP3^+^ Treg clones (mean RFI=21.19 ± 2.08 and 46.29 ± 9.80 for blood- and LPL-derived clones, respectively) (**Figures 1E, F**). Furthermore, DP8α clones secreted IFNγ (**Figures 1A, B**) and IL-10 (**Figures 1C, D**) upon stimulation. A fraction of FoxP3^+^ Treg clones also secreted IL-10, however, none of these clones secreted significant levels of IFNγ, which when co-produced with IL-10 represents a feature of Tr1-like Tregs (10). All DP8α clones expressed the purinergic receptors CD39 and CD73 (**Figures 1G, H**), and blocking these enzymes neutralized their ability to inhibit effector T cell proliferation (**Figures 1I, J**). In contrast, whereas some FoxP3^+^ Treg clones also expressed CD39, they expressed low to no CD73 (**Figures 1G, H**), and their inhibition of effector T cell proliferation was unaltered following CD39 and CD73 inhibition (**Figures 1I, J**). Therefore, DP8α regulatory function, unlike FoxP3^+^ Treg function, depends on the purinergic pathway. Similar to clone data, *ex vivo* analyzed circulating DP8α Tregs systematically expressed CD39 and CD73 in all donors, whereas CD39 was expressed by blood FoxP3^+^ Tregs in only two donors, and CD73 in none of them (**Figure 1K**).

### Transcriptomic comparison of human DP8α and FoxP3^+^ Treg clones isolated from the colon lamina propria and blood

**Figure 2:**
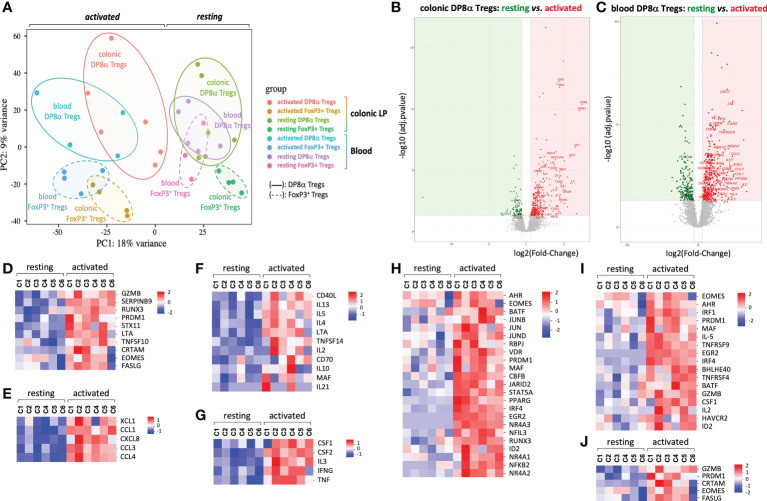
Principal component and DEG analyses. Transcriptomic comparison of human DP8α and FoxP3^+^ Treg clones isolated from the colonic lamina propria and blood from 4-6 subjects, activated or not. **(A)** Principal component analysis. **(B**, **C)** Log2 fold-change of average expression values for genes expressed by resting *versus* activated DP8α clones: colon-derived **(B)** blood derived **(C)**. **(D-I)** Heat-maps showing normalized expression of selected DEGs between resting and activated colon-derived DP8α clones. Genes involved in cytotoxicity **(D)**, encoding chemokines **(E)**, involved in B cell help **(F)**, encoding cytokines **(G)**, encoding TF **(H)**, genes of the Tr1 signature **(I)** and genes corresponding to a CD4-CTL signature **(J)** are represented.

As shown by Principal Component Analysis (PCA), DP8α clones, whether they were derived from PBLs or LPLs, clustered apart from FoxP3^+^ clones (**Figure 2A**). At variance, DP8α clones of blood and colonic origin, despite their dispersion, clustered in the same area at the resting state and, to some extent, also upon activation, Moreover, no differentially expressed gene (DEG) distinguished resting colon- and blood-derived DP8α clones and only 63 were identified upon activation, underlying the robust relationship between colon- and blood-derived DP8α Tregs (not shown).

In contrast, LPL-derived and PBL-derived FoxP3^+^ clones clustered apart by PCA (**Figure 2A**), indicating significant differences between these cells according to their location. Nonetheless, the number of DEGs between these clones was low both at the resting state (n=98, up: 47, down: 51) and after activation (n=230, up: 99, down: 131). This may result from a significant heterogeneity of colonic FoxP3^+^ Tregs likely due, in part, to the co-existence in this compartment, of tissue-resident Tregs and central memory Tregs circulating through colonic lymphoid structures. Analysis of individual FoxP3^+^ Treg clone transcriptomes and cytokine profiles indeed revealed a duality within colon-derived FoxP3^+^ clone profiles (**Figure S2**), particularly for CD62L transcripts (*SELL*) expression, a lymphoid tissue related gene.

### Activation-induced transcriptomic signature of DP8α Tregs

We then investigated the activation-induced responses of DP8α Treg clones through DESeq2 analysis of 3’SRP transcriptomic data obtained in activated *versus* resting colon- and blood-derived DP8α clones. Applying the criteria for significance (FDR < 0.05 and absolute value of log2 fold-change > 0.5), 323 and 379 genes were significantly up-regulated and 93 and 208 genes down-regulated upon activation, in colon- and blood-derived DP8α clones, respectively (**Figures 2B, C**). We first focused on the 102 genes up-regulated in activated colon-derived DP8α clones, for which roles in T cells had already been documented.

Strikingly, ten genes clearly conveyed cytotoxic functions. The class-I-restricted cytotoxic associated molecule, *CRTAM*, known as an inducer of cytotoxic activity and IFNγ production in CD4^+^ T cells (11), transcription factors (TFs) involved in cytotoxic cell differentiation, *EOMES*, *PRDM1* and *RUNX3*, genes encoding cytotoxic cytokines or receptors: *FASLG*, *TNFSF10* (or *TRAIL*), *GZMB* and *LTA*, as well as *STX11* which facilitates cytolytic granule exocytosis (12) and *SERPINB9* (**Figure 2D** and **Figure S3**), known to protect GZMB-producing Tregs from lysis (13).

Six genes encoded chemokines: CCL1, which attracts Th2 lymphocytes *via* CCR8; CCL3 and CCL4, which recruit cells expressing CCR1 and/or CCR5, essentially T cells (14); XCL1, which acts specifically on antigen cross-presenting dendritic cells (15); CXCL8 (IL-8) (**Figure 2E**), a chemokine which can recruit neutrophils (16) and CKLF (**Figure S4**), which attracts CCR4-expressing targets such as Th2 cells (17).

CD3-mediated activation also up-regulated numerous genes encoding molecules known to provide help to B cells and/or drive antibody class-switch recombination or secretion, such as *CD40LG*, *IL10*, *IL4*, *IL21* (18), *CD70*, *IL2*, *IL13*, *IL5*, *LTA*, *MAF*, *TNFSF14* (or *LIGHT*) (**Figure 2F** and **Figure S5**), as well as genes encoding additional cytokines or growth factors (**Figure 2G**).

Particular TF combinations were reported to characterize homogeneous cell subsets more accurately than surface markers. Interestingly, 23 TFs, including those mentioned above, were induced or up-regulated by DP8α Tregs upon activation (**Figure 2H** and **Figure S6**). Strikingly, all these TFs have been reported to play roles in the differentiation, homeostasis or suppressive functions of Tregs. This is the case for *EOMES*, which together with *PRDM1* (or *BLIMP1*), induces IL-10 expression and Tr1-like cell differentiation (19, 20), *PRDM1* also being a crucial regulator for microbiota-induced murine FoxP3^+^/RORγt^+^ Tregs (21, 22); *AHR*, that marks and promotes gut-derived Tregs and positively regulates IL-10 and Tr1 differentiation (23); *CBFB*, a master regulator of genes involved in the induction of FoxP3^+^ Tregs and their suppressive functions (24); *EGR2*, a Tr1-associated TF (25); *ID2*, *IRF4*, *BATF* and *PPARG*, which orchestrate the development and maintenance of adipose tissue Tregs (26); *IRF1*, which together with *BATF* is required for the development of IL-27-induced Tr1 cells (27); and *MAF*, a key regulator of intestinal immune homeostasis through sustaining RORγt expression in mouse microbiota-induced gut Tregs and positively regulating IL-10 production (21, 22).

Interestingly, consistent with the Tr1-like properties of DP8α Treg clones detailed above, *i.e.*, mainly the lack of FoxP3 expression together with the secretion of both IL-10 and IFNγ, DP8α Tregs significantly shared features with either human or mouse Tr1 and Tr1-like signatures, namely *GZMB*, *CSF1*, *HAVCR2* (or *TIM3*), *EGR2*, *PRDM1*, *IRF4*, *MAF*, *EOMES*, *AHR*, *IRF1*, *BATF*, *ID2*, *BHLHE40*, *TNFRSF9* (or *41BB*), *TNFRSF4* (or *OX40*), *IL-5*, *IL-2* ^LOW^ (19, 20, 28–32) (**Figure 2I**), as well as *GZMK* (31, 32), identified in DP8α Treg clones as down-regulated upon activation (**Figure S7**). Moreover, all activated DP8α Treg clones but one also expressed *CRTAM* (**Figure 2J**), which together with *EOMES*, *FASLG*, *GZMB* and *PRDM1* represent a cytotoxic CD4^+^ effector cell signature (11).

Further supporting the chemotactic and lytic potentials of DP8α Tregs, *CCL5*, *CCR5*, *GZMA* and *GZMK* were expressed by resting DP8α clones and down-regulated but still highly expressed upon activation (**Figure S7**).

Regarding blood-derived *F. prausnitzii*-reactive DP8α Treg clones, most genes up-regulated by activation overlapped those up-regulated in colon-derived DP8α Treg clones (**Figure 2C**), although some of these alterations remained statistically unsignificant, likely due to the low number of studied clones. This result, together with the lack of DEG between blood- and colon-derived clones (not shown), strengthens the notion that DP8α cells found in both compartments are identical and originate from the gut.

### Identification of a core gene signature differentiating DP8α from FoxP3^+^ human Tregs

**Figure 3:**
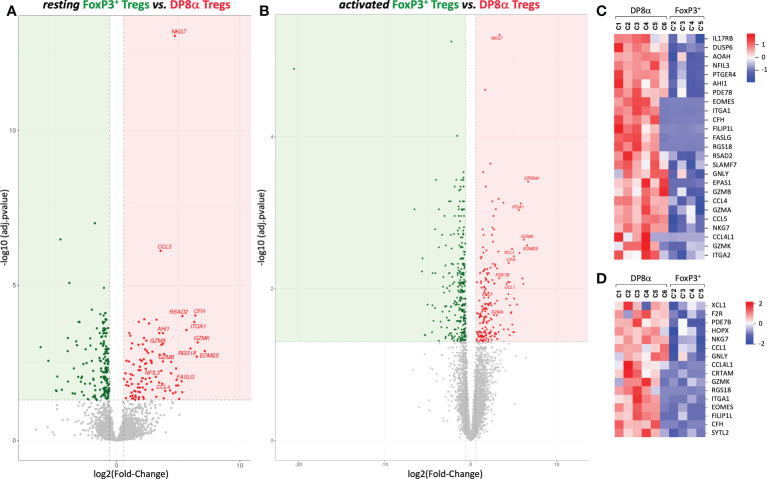
Gene expression profiles generated from DP8α and FoxP3^+^ Treg clones derived from the colonic lamina propria from patients and healthy donors. **(A**, **B)** The Log 2-fold changes of average expression values between resting **(A)** or activated **(B)** DP8α and FoxP3^+^ clones. **(C**, **D)** Heat-maps showing normalized expression of top DEGs between resting **(C)** or activated **(D)** colon-derived DP8α and FoxP3**^+^** Treg clones.

We then compared DP8α to canonical FoxP3^+^ Treg clones derived from the same compartments, *i.e.*, blood or colonic lamina propria, though DESeq2 analysis. Importantly, despite the limits mentioned above regarding the low number and heterogeneity of FoxP3^+^ clones, colon-derived DP8α and FoxP3^+^ Treg clones clearly diverged by PCA (**Figure 2A**) as well as by 286 (up: 108, down: 178) and 506 (up: 199, down: 307) DEGs at the resting and activated states, respectively (**Figures 3A, B**).

In contrast, blood-derived DP8α and FoxP3^+^ clones differed by a limited number of DEGs (**Figure S8**). However, examination of unfiltered results revealed that most up-regulated DEGs identified in colon-derived DP8α *versus* FoxP3^+^ clones were also expressed at higher levels in DP8α, as compared to FoxP3^+^ blood-derived clones, as shown by log2 fold-changes > 1. Nonetheless, most of these differences remained statistically non-significant (**Figure S8**), likely due to the low number and heterogeneity of both PBL-derived Treg clone subsets.

We next focused on DEGs identified between colon-derived DP8α and FoxP3^+^ Tregs. Top up-regulated DEGs identified in resting and activated DP8α *versus* FoxP3^+^ Treg clones (log2 fold-change ranging from 3 to 6.6) are represented (**Figures 3C, D**), respectively. As mentioned above, the expression of *EOMES* together with *GZMK*, *GZMA*, *GZMB* and *CRTAM* are hallmarks of cytotoxic CD4^+^ T cells (11, 33), while the co-expression of *EOMES*, *GZMK* and *ITGA2* targets Tr1-like cells (31, 32). Other salient features of top DP8α Treg DEGs were a substantial prevalence of genes that 1/define a cytotoxic profile, such as *FASLG*, *GZMA*, *GZMB*, *GZMK*, *GNLY*, *NKG7*, *SLAMF7*, and *SYTL2, 2*/encode chemokines, including *CCL1*, *CCL4*, *CCL4L1*, *CCL5* and *XCL1*, and 3/encode TFs reported to inhibit FoxP3^+^ expression or FoxP3^+^ Treg differentiation: *EOMES* (34), *EPAS1* (35) and *NFIL3* (36).

Altogether, these data showed that DP8α Tregs have a highly differentiated transcriptomic signature evocative of major regulatory functions *via* cytotoxicity as well as chemotaxis through both chemokine secretion facilitating their suppressive interaction with effector T cells and DCs, and robust expression of chemokine receptors, such as *CCR1*, and *CCR5* (at steady state), favoring migration towards inflamed tissues.

### DP8α Tregs, but not FoxP3^+^ Tregs, efficiently kill cells of myeloid origin

**Figure 4:**
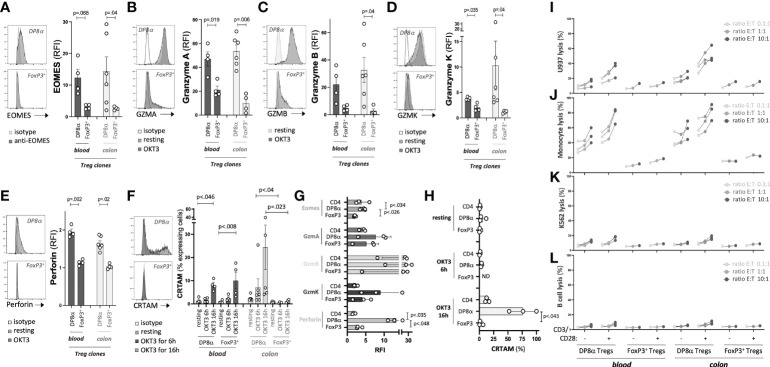
DP8α Tregs can kill myeloid cells. **(A**, **F)** Clone expression of EOMES **(A)** Granzyme A **(B)** Granzyme B **(C)** Granzyme K **(D)** Perforin **(E)** measured by intracellular labelling following or not activation for 6h and cell surface expression of CRTAM **(F)** following or not activation for the indicated time. Clones were activated with coated 0.5μg/ml OKT3. **(G, H)** PBMCs from 3 HD were OKT3-stimulated for either 6h and stained intracellularly for EOMES, Granzymes A, B, K and Perforin **(G)** or 6h and 16h and stained for CRTAM **(H)**. Expression of these proteins is shown for indicated T cell subsets. **(I-L)** Indicated target cells were co-cultured with blood- and colon-derived DP8α and FoxP3^+^ Treg clones (stimulated or not with CD3/CD28 beads) at indicated ratios. Cell death was measured as described in the Methods’ section. Paired 2-sided t-tests were used for single comparisons, while one-way ANOVA followed by Dunn’s multiple comparison tests were performed for 4F-H, p<.05 was considered statistically significant.

Since clones were used to characterize the transcriptome of DP8α Tregs, the expression of molecules of interest as well as their *in vitro* function could be assessed using the exact same cells. First, based on the unique prevalence of cytotoxicity-associated genes in the transcriptome of DP8α Tregs and the significant overlap of the DP8α Treg signature with the Tr1 one, we measured the expression of such proteins by Treg clones, and their ability to kill cells of myeloid origin, which are typical targets of Tr1 cells (19, 37). In accordance with DEG data, DP8α Treg clones expressed EOMES protein, in contrast with FoxP3^+^ Treg clones (**Figure 4A**), as well as GZMA (**Figure 4B**), GZMB (**Figure 4C**), GZMK (**Figure 4D**) and Perforin (**Figure 4E**) at higher levels than FoxP3^+^ Treg clones did. Moreover, colon-derived DP8α Treg clones significantly expressed CRTAM shortly after activation (6h), which further increased at 16h post-activation, whereas colon-derived FoxP3^+^ Treg clones did not, regardless of the duration. At variance, CRTAM induction appeared delayed and was detected only at the 16h time-point in blood-derived DP8α clones. Moreover, CRTAM was also expressed by two blood-derived FoxP3^+^ Treg clones (**Figure 4F**), which, to our knowledge, has not been described so far. Similarly, in PBMCs, these molecules were all over-expressed by CCR6^+^/CXCR6^+^ DP8α Tregs, as compared to total CD4^+^ T cells or CD4^+^/CD25^HIGH^/CD127^LOW^ classical FoxP3^+^ Tregs (**Figures 4G, H**).

To assess the ability of DP8α Tregs to kill myeloid cells, two cell lines, namely K562, of the erythroleukemia type, and U937, of the myeloid lineage, as well as two types of primary *ex vivo* sorted cells, CD19^+^ B cells and CD14^+^ monocytes, were co-cultured with Treg clones. No cell lysis was observed at 4h, timing usually required by conventional CD8^+^ T cells to exert cytotoxicity (not shown). In contrast, at 16h, potent lysis activity against cells of myeloid origin (U937 and monocytes) was induced by activated DP8α Tregs and, to a lesser extent, even non-stimulated DP8α Tregs, whether they were derived from blood or colon (**Figures 4I, J**). DP8α Treg clones neither significantly killed K562 nor sorted B cells (**Figures 4K, L**). No FoxP3^+^ Treg clone exerted any significant cytotoxicity (**Figures 4I-L**).

Therefore, in accordance with their expression of numerous molecules associated with a cytotoxic activity, DP8α Tregs unambiguously exert actual potent cytotoxic functions against myeloid cells, as reported for Tr1 cells (19, 37).

### DP8α Tregs promote B cell differentiation towards IgA-secreting B cells

In mice, colonic IL-10-secreting Foxp3^+^ Tregs are thought to play a role in the maintenance of a healthy gut microbiota by regulating the production of microbiota-reactive secretory IgA (21).

**Figure 5:**
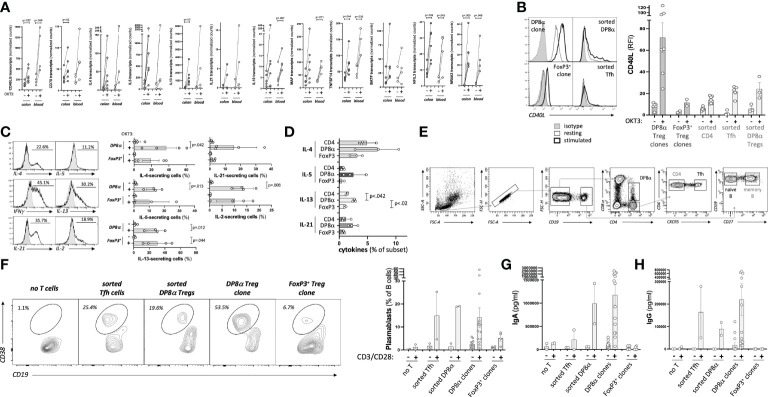
Blood-derived DP8α Tregs promote IgA-secreting B cell differentiation. **(A)** Expression by DP8α Treg clones of transcripts involved in B cell help. **(B)** CD40L: representative histogram plots examples (left) and RFI for all tested cell types (right). **(C)** Cytokine production by DP8α clones: representative histogram plots examples (left) and all clone data (right) are shown. **(D)** PBMCs from 3 HD were OKT3-stimulated and stained for indicated cytokines. **(E)** Gating strategy used for the sortings of T and B cell subsets. **(F-H)** Sorted naïve B cells were co-cultured with autologous indicated CD4^+^ T cell subsets or Treg clones (ratio 1:1). Plasmablast frequency was assessed 10 days later **(F)**. Supernatants were tested for total IgA **(G)** and IgG **(H)** contents. Paired 2-sided t-tests were used for single comparisons, while one-way ANOVA followed by Dunn’s multiple comparison tests were performed for 5D, p<.05 was considered statistically significant.

Since the present study identified an array of genes involved in B cell help expressed by DP8α Tregs upon activation (**Figure 5A**), we asked whether blood-derived DP8α Tregs (*ex vivo* sorted and clones) could indeed provide B cell help and induce IgA production *in vitro*, as compared to both FoxP3^+^ Treg clones and sorted follicular helper T (Tfh) cells.

First, the expression of key proteins known to affect B cell activation and differentiation was analyzed. CD40L was strongly expressed by DP8α Treg clones, even at the steady state, and, to a lower extent, by *ex vivo* sorted DP8α Tregs, similar to Tfh cells used as a positive control, while Foxp3^+^ Treg clones barely did (**Figure 5B**). Production of several cytokines known to also influence B cell development and functions by both blood-derived clones and PBMCs was tested. Significant fractions of DP8α and FoxP3^+^ Treg clones did produce IL-2, IL-4, IL-5, IL-13 and IL-21 (**Figure 5C**) in addition to IL-10 (**Figures 1C, D**) and IFNγ (**Figures 1A, B**). Moreover, OKT3-stimulated PBMCs showed that DP8α Tregs produced higher levels of these cytokines than classical Tregs did (**Figure 5D**), demonstrating that DP8α Tregs are well-equipped to provide B cell help.

To directly determine whether DP8α Tregs can promote B cell activation and differentiation, clonal or *ex vivo* sorted DP8α cells were co-cultured with *ex vivo* sorted naïve B cells in the presence of an agonistic anti-BCR antibody with or without CD3/CD28 beads. As controls, *ex vivo* sorted Tfh cells and Foxp3^+^ Treg clones were identically run alongside. The gating strategy to sort these various T and B cell subsets is represented (**Figure 5E**). Ten days later, plasmablasts (CD3^-^/CD4^-^/CD19^LOW^/CD38^+^) were detected when naïve B cells had been co-cultured with DP8α Tregs (**Figure 5F**). Plasmablasts represented a mean of 19.1% ± 0.21 and 14.1% ± 4.21 (ranging from 0.8 to 56.9%) of total CD3^-^/CD4^-^/CD19^+^ B cells after co-culture with activated sorted autologous DP8α Tregs and DP8α Treg clones, respectively (**Figure 5F**). It is noteworthy that DP8α Treg clones are heterogenous, *i.e.*, while some induced high percentages of plasmablasts, other induced only low to no B cell differentiation. This plasmablast induction was comparable to that obtained in the presence of activated Tfh cells, while it was much lower (5.2% ± 1.39 of total B cells) when Foxp3^+^ Treg clones were used (**Figure 5F**).

In parallel, supernatants were tested for total IgA and IgG contents. Blood-derived DP8α Tregs, both clonal and sorted polyclonal cells, induced high levels of IgA production (1177ng/ml ± 219.3 and 990ng/ml ± 433.4, respectively), seemingly above levels induced by autologous Tfh cells (211ng/ml ± 210) (**Figure 5G**). DP8α Tregs also induced IgG production (90ng/ml ± 28.5 and 220ng/ml ± 71.8 for sorted and clonal cells, respectively) at similar levels than Tfh cells (164ng/ml ± 114.3) did (**Figure 5H**). Of note, Foxp3^+^ Tregs did not induce IgA nor IgG production (**Figures 5G, H**).

Therefore, DP8α Tregs do provide B cell help and seem prone to promote IgA class switch recombination.

### Uniquely high chemotactic potential of DP8α Tregs

**Figure 6:**
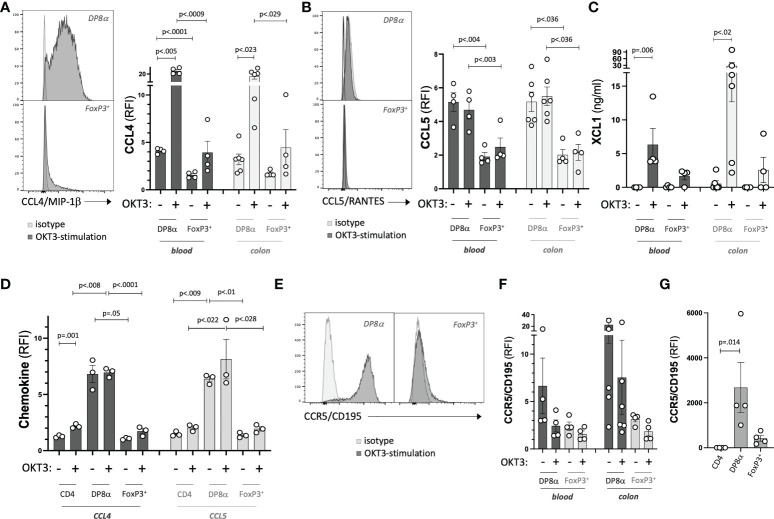
Chemotactic potential of DP8α Tregs. **(A-C)** Expression of indicated chemokines by representative Treg clones (left) and all studied Treg clones (right). **(D)** Expression of CCL4 and CCL5 by PBMCs from 3 HD shown for indicated gated T cell subsets. **(E-G)** Expression of CCR5 by representative Treg clones **(E)**, all Treg clones **(F)** and gated subsets from PBMCs **(G)**. Paired 2-sided t-tests were used for single comparisons, while one-way ANOVA followed by Dunn’s multiple comparison tests were performed for 6A-D,G, p<.05 was considered statistically significant.

Next, to formally establish the chemotactic potential of DP8α Tregs, we measured the protein expression of genes involved in this function, namely CCL4, CCL5, XCL1 and CCR5. In accordance with their differential transcription, all three chemokines were expressed at significantly higher levels by DP8α Treg clones, both colon- and blood-derived, as compared to FoxP3^+^ Treg clones derived from same compartments (**Figures 6A-C**), suggesting a significant ability of both circulating and colon-resident DP8α Tregs to recruit target cells. Strikingly, intracellular expression of CCL4 and CCL5 proteins by circulating DP8α Tregs was also detected *ex vivo*, clearly discriminating these cells from circulating FoxP3^+^ Tregs, at both resting and activated states (**Figure 6D**).

Also relevant in term of DP8α Treg regulatory potential, through recruitment to inflammatory sites (38), CCR5 expression levels were higher in resting colon- and blood-derived DP8α clones, as compared to matched FoxP3^+^ clones (**Figures 6E, F**), a difference confirmed *ex vivo* (**Figure 6G**).

Therefore, DP8α Tregs appear well-equipped to both recruit target cells and be recruited towards appropriate sites, especially when compared to classical FoxP3^+^ Tregs.

### DP8α Tregs express RORγt

**Figure 7:**
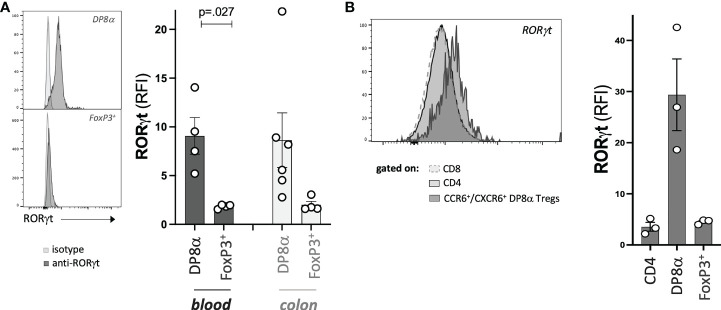
DP8α Tregs express RORγt. Expression of RORγt by representative Treg clones (left) and all Treg clones (right) **(A)**, as well as indicated gated subsets from PBMCs **(B)**. Paired 2-sided t-tests were used for single comparisons, while one-way ANOVA followed by Dunn’s multiple comparison tests were performed for 7B, p<.05 was considered statistically significant.

Finally, we assessed the protein expression by DP8α and FoxP3^+^ Treg cells of the RORγt TF, known to characterize microbiota-induced mouse Tregs (21, 22). In a previous study (6), we had reported the expression of RORγt by a few blood-derived DP8α Treg clones not studied here. Here, since this gene was not detected through the 3’seq RNA profiling method used, we assessed its expression by all studied Treg clones and their circulating counterparts. RORγt was expressed at the resting and activated states, by both blood- and colon-derived DP8α Treg clones, while it was not by FoxP3^+^ Treg clones (**Figure 7A**). Moreover, strikingly, this TF was also expressed by circulating DP8α Tregs among PBMCs, but not by other T cell subsets, such as total CD4^+^, classical CD25^HIGH^/CD127^LOW^ Tregs, CD8^+^, as well as double positive CD4^+^/CD8^+^ T cells lacking CCR6 and CXCR6 co-expression, which do not respond to *F. prausnitzii* (**Figure 7B** and not shown).

Therefore, RORγt represents yet another marker, which expression is preferably restricted to *F. prausnitzii*-induced DP8α Tregs.

## Discussion

Here, we identified the signature for human *F. prausnitzii*-specific DP8α Tregs as partly overlapping with those described for both Tr1-like Tregs (**Figure 2I**) and, more surprisingly, cytotoxic CD4 T cells (CD4-CTL) (**Figure 4** and **Figure S3**), clearly establishing the uniqueness of these Tregs. Moreover, we comforted that blood- and colon-derived DP8α Tregs were quasi-undistinguishable, supporting their common origin (**Figure 2**), as previously suggested by the simultaneous decrease of these cells in both compartments in IBD patients (3, 6). Importantly, their presence in blood further advocates for systemic roles of *F. prausnitzii*-specific DP8α Tregs.

In mice, RORγt^+^ FoxP3^+^ intestinal Tregs were shown to develop against microbial antigens and have been proposed to be specialized in maintaining tolerance to the gut microbiota, in a cMAF dependent manner, through the control of Th17 responses (22). Moreover, they also play roles in immune homeostasis and health beyond the intestine, such as preventing allergy and as a defect in their induction at weaning leads to increased susceptibility to metabolic and chronic inflammatory diseases later in life (1). We revealed here that, besides their induction by related *Clostridium* bacteria and preferential location within the colonic LP, human DP8α Tregs also share with mouse microbiota-induced intestinal FoxP3^+^ Tregs, the expression of the lineage-defining TFs, RORγt (**Figure 7**) and MAF (**Figures 2F, H, I**). This developmental resemblance between both subsets suggests that DP8α Tregs could represent human functional counterparts of mouse intestinal RORγt^+^/FoxP3^+^ Tregs, which prevent colitis. Strengthening this hypothesis, we recently demonstrated that infusion of a DP8α Treg clone, together with *F. prausnitzii* gavage, protected NSG mice humanized for the Treg HLA class-II restricting allele, against intestinal inflammation (7), which, together with the drastic and specific decrease of these cells in IBD patients, strongly support their role in colon homeostasis. Surprisingly, while RORγt expression characterizes microbiota-induced mouse FoxP3^+^ Tregs, we failed to detect RORγt-expressing cells by flow cytometry among both the FoxP3^+^ clones (colon- and blood-derived) and PBMC-derived CD3^+^/CD4^+^/CD25^HIGH^/CD127^LOW^ T cells (**Figures 7A, B**).

Remarkably, even though various human gut microbes, especially commensals, promoting colonic FoxP3^+^ Treg differentiation in mice have been identified (39–43), to our knowledge, direct evidences regarding the presence of Tregs specific for these bacteria in the human colonic mucosa, either FoxP3^+^ or Tr1-like, are still lacking. Nonetheless, the presence of commensal-specific T cells has been shown, among both circulating CD4^+^ effectors T cells (44) and CD4^+^/CD8αα^+^ regulatory intra-epithelial lymphocytes in the small intestine (45), both populations being key in human gut barrier integrity maintenance.

Our transcriptomic and protein data also revealed that activated DP8α Tregs expressed a large panel of molecules involved in cytotoxicity (**Figure 2D**, **Figure S3** and **Figures 4A-H**), at various levels: direct cytotoxicity, degranulation and self-killing prevention. In accordance, we showed the potent ability of DP8α clones to specifically kill myeloid cells *in vitro* (**Figures 4I-L**), a property proposed to be an immunosuppressive mechanism for Tr1 cells through antigen-presenting cells killing, hence limiting subsequent T cell priming/activation (19). Strikingly, we also showed that DP8α Tregs secreted XCL1 (**Figure 6C**), which targets XCR1a-expressing DCs, hence could promote DP8α Treg-DC contacts and DC killing.

Supporting their high trafficking potential, resting DP8α clones and *ex vivo* circulating DP8α cells expressed high levels of CCR5 (**Figures 5E-G**), which supports migration to inflammatory sites (38). These cells also produced a large array of chemokines, *e.g.*, CCL1, CCL3, CCL4, CCL5 and XCL1 (**Figure S4** and **Figures 6A-D**). This unveils their strong potential to recruit various target cells, such a CCR8-, CCR1- and CCR5-expressing effector T cells, as well as bystander CCR5^+^ DP8α Tregs (**Figures 6E-G**). CCL3 and CCL4 are known to be expressed by classical FoxP3^+^ Tregs (14) and CCL5 by a unique blood-derived Treg subset (46). Moreover, through XCL1 secretion, DP8α Tregs should efficiently interact with XCR1a-expressing cells, reported to be the human cross-presenting BDCA3+ DC subset (15). Therefore, these interactions should be key in the ability of DP8α Tregs to dampen the proliferation (6) and cytokine secretion (unpublished personal data) of effector cells, as shown *in vitro.* In addition, the XCL1-XCR1-mediated interactions, shown in mice to be stable (15), could extend DP8α Treg-DC interactions, thus promoting either DC-killing or inhibition of DC maturation in a CTLA-4-dependent manner, as reported previously (3), or merely restraining DCs from Tconv. All these effects could be implicated in establishing and/or restoring local tolerance against gut microbiota.

Based on their IL-10 and IFNγ production (**Figures 1A-D**) as well as their lack of stable FoxP3 expression (**Figures 1E, F**), DP8α cells partly resemble Tr1-like Tregs. Our data identified additional similarities between DP8α Tregs and this highly heterogeneous Treg subset (**Figure 2I** and **Figure S7**), such as the expression of the ITGA2 Tr1 marker and cytotoxicity towards myeloid cells (19). Nonetheless, we also provided evidences that DP8α Tregs clearly differed from Tr1 cells. For instance, DP8α cells produced IL-4 (**Figures 5A, C, D**), not expressed by Tr1 cells, and conversely lacked TGFβ transcription and PD-1 expression (not shown), both considered critical for Tr1-mediated suppression (19). Moreover, effector-inhibition by DP8α Tregs strongly relied on CD39- and CD73-mediated metabolic disruption *in vitro* (**Figures 1I, J**), and likely on their high chemotactic potential *in vivo*, two properties not documented for conventional Tr1 cells. Finally, while the exact origin and stability of Tr1 and Tr1-like Tregs remains questionable (32), DP8α Tregs do represent stable components of healthy donor blood and colonic lymphocytes, therefore embodying a unique physiologically-induced colon-derived Treg subset.

Early reports in mice demonstrated a supportive role of Treg cells for intestinal IgA production, possibly due to their plasticity to differentiate into Tfh or Tfr cells or to secrete IL-10 (21). Here, we showed that *ex vivo*-sorted DP8α cells and DP8α clones induced naïve B cells to differentiate into plasmablasts as efficiently as circulating Tfh did (**Figure 5F**) and induced seemingly higher levels of IgA production (**Figure 5G**), adding yet another shared function with intestinal mouse FoxP3^+^ Tregs. Of note, FoxP3^+^ Treg clones did not induce Ig-producing plasmablasts (**Figures 5F-H**). Therefore, DP8α Tregs do provide B cell help and seem particularly prone to promote IgA class-switch recombination. This function could be key in the gut, where these cells are fairly represented (>10% of total LP CD4^+^ T cells (3, 5, 6)), to maintain homeostasis in an IgA-dependent manner. Mechanisms involved in IgA-induction by DP8α Tregs, as well as their compatibility with the double-negative feedback loop between RORγt^+^ intestinal Tregs and IgA production reported in mice (47), remain to be elucidated.

The class I-restricted associated molecule (CRTAM) was originally described as transiently induced upon activation on cytotoxic CD8^+^ and NK cells. Recently, it has been reported to drive the CD4-CTL lineage, together with MAF, GZMK and EOMES (11, 33). Here, we showed that DP8α Tregs expressed these molecules (**Figure 4** and **Figure S3**).

Effector targets of DP8α Tregs remain to be identified. Since many studies established that specialized Treg subsets acquire part of the transcriptional profile of their effector cell counterparts (48), the present demonstration that DP8α Tregs express RORγt and CRTAM, the canonical transcription factor of Th17 and cytotoxic CD4, respectively, suggests that these effectors could be their specific targets. Indeed, mouse colonic RORγt-expressing microbiota-induced Tregs were shown to inhibit Th17-mediated inflammation (21). Moreover, CD4-CTL were reported to be enriched in the mouse intestinal mucosa, as compared to secondary lymphoid organs, and to promote experimental colitis (11). Although not yet reported, the presence of CD4-CTL in the human intestinal mucosa appears conceivable as their differentiation is restrained by histone deacetylases (49), which inhibitors, such as butyrate, are particularly abundant in this compartment.

While the use of well characterized clones for the present genomic study allowed to both analyze a relatively homogeneous population of DP8α Tregs and, importantly, perform phenotypic and functional studies with the very same cells, the limit of this approach is that it could not address the full potential heterogeneity of *F. prausnitzii*-reactive DP8α cells *in vivo* in health and diseases. This question should be addressed through single-cell RNA-seq analyses using the method of simultaneous epitope and transcriptome measurement in single cells (50). Nonetheless, it has to be stressed that tested markers and functions identified using clones were corroborated using *ex vivo* PBMC-derived DP8α Tregs, indicating both their relevancy and that they were not related to clone sorting and/or long-term culture.

While the developmental origin of the many described Tr1-like cells remains elusive, DP8α cells likely differentiate into Treg cells due to their specificity. Indeed, we demonstrated that *F. prausnitzii* harnessed dendritic cell functions and rendered them tolerogenic in a TLR2/TLR6-dependent manner^4^. As a consequence, *F. prausnitzii*-derived epitopes presented by dendritic cells end-up priming naïve CD4 T cells into IL-10-producing CD4^+^ T cells^4^, very likely including DP8α Tregs.

To conclude, this study brought forward key new data regarding the transcriptomic signature, phenotype and functional properties of DP8α Tregs, namely their potent lytic activity, chemotactic potential and their unique ability to promote IgA production. These features clearly single out this subset from FoxP3^+^ Tregs as demonstrated here, but also from Tr1-like cells, as described above, for instance in terms of specific functions and physiological relevance. Instead, they rather seem closest to mouse RORγt^+^/FoxP3^+^ gut Tregs, demonstrated to protect against colitis (2). Altogether, these findings strongly suggest that these unique microbiota-induced Tregs represent previously underappreciated crucial actors of tissue homeostasis, emphasizing the importance to further explore their roles in health and diseases, particularly in the context of IBD.

## Data availability statement

The datasets presented in this study can be found in online repositories. The name of the repository and accession number can be found below: NCBI Gene Expression Omnibus; GSE197906.

## Ethics statement

The studies involving human participants were reviewed and approved by CPP Ouest IV-Nantes institutional board of Nantes University Hospital. The patients/participants provided their written informed consent to participate in this study.

## Author contributions

FJ analyzed RNA sequencing data, co-designed and interpreted experiments as well as wrote the manuscript; JA produced and screened LPL-derived clones; RT performed bioinformatic analyses and transcriptomics data illustrations; AP performed part of the T/B co-culture experiments; NJ helped design and perform flow cytometry-based sortings and clonings; FA supervised the project; EG designed and performed most experiments as well as supervised the project; EG, FA, and FJ edited the manuscript. All authors contributed to the article and approved the submitted version.

## Funding

This work was supported by ANR (MICITREG, ANR-15-CE17-0010).

## Acknowledgments

We thank the members of INCIT INSERM unit 1302 (Immunology and New Concepts in ImmunoTherapy), with a special emphasis for members of Frédéric Altare’s laboratory for technical help and discussions. We also thank Dr Nathalie Labarrière for her kind gift of the K562 and U937 cell lines. We thank Julie Tabiasco for providing us with the CD39 inhibitor POM-1. We are most grateful to the Genomics Core facility *GenoA*, member of Biogenouest and France Genomique and to the Bioinformatics Core facility *BIRD*, member of Biogenouest and Institut Français de Bioinformatique (IFB) (ANR-11-INBS-0013) for the use of their resources and technical support. We thank Harry Sokol and Chantal Bridonneau for providing us the A2-165 *Faecalibacterium prausnitzii* bacterium. We acknowledge the contribution of the staff of Cytocell (Flow cytometry and FACS core facility, BioCore, CNRS 3556 - Inserm US016 - Nantes Université UMS 3556, Nantes) for their expert technical assistance with flow cytometry material and methods.

## Conflict of interest

The authors declare that the research was conducted in the absence of any commercial or financial relationships that could be construed as a potential conflict of interest.

## Publisher’s note

All claims expressed in this article are solely those of the authors and do not necessarily represent those of their affiliated organizations, or those of the publisher, the editors and the reviewers. Any product that may be evaluated in this article, or claim that may be made by its manufacturer, is not guaranteed or endorsed by the publisher.
