# Supplementary material for: Human gut microbiota-reactive DP8α regulatory T cells, signature and related emerging functions
Source: Front Immunol. 2022 Nov 21;13:1026994. doi: 10.3389/fimmu.2022.1026994 (PMC9720269; doi:10.3389/fimmu.2022.1026994)
Supplement: Supplementary file 1 [file DataSheet_1.pdf]

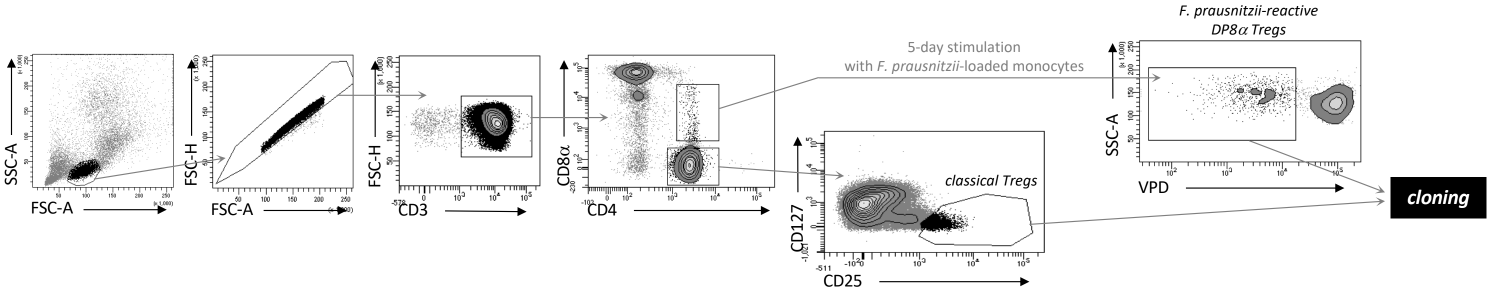

Figure S1. **Production of human Treg clones.** Purified VPD-stained $CD4^{+}$ T cells, comprising DP8α Tregs, were co-cultured with purified autologous $CD14^{+}$ monocytes loaded overnight with *F. prausnitzii* (1:1 ratio). Five days later, *F. prausnitzii*-reactive VPD^{LOW} $CD3^{+}/CD4^{+}/CD8\alpha^{LOW}$ cells were cloned from 4 healthy donors using the Aria III cell sorter. Donor-matched $CD3^{+}/CD4^{+}/CD8^{-}/CD25^{HIGH}/CD127^{LOW}$ classical Tregs were also cloned. DP8α Tregs and $FoxP3^{+}$ Tregs were similarly cloned from freshly-dissociated, sorted colonic LPLs. All clones were then amplified on feeder cells[4] for at least 4 weeks, before they were used for the present study.

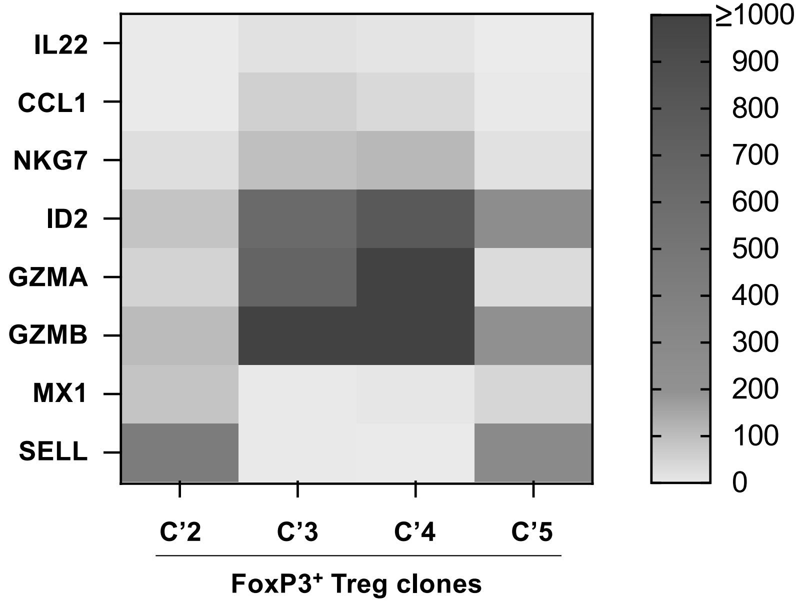

Figure S2. **Transcript expression heterogeneity by FoxP3+ Treg clones derived from the colonic lamina propria.** Normalized counts for indicated transcripts expressed by OKT3-stimulated individual FoxP3+ Treg clones derived from the healthy colonic lamina propria are represented as a heatmap.

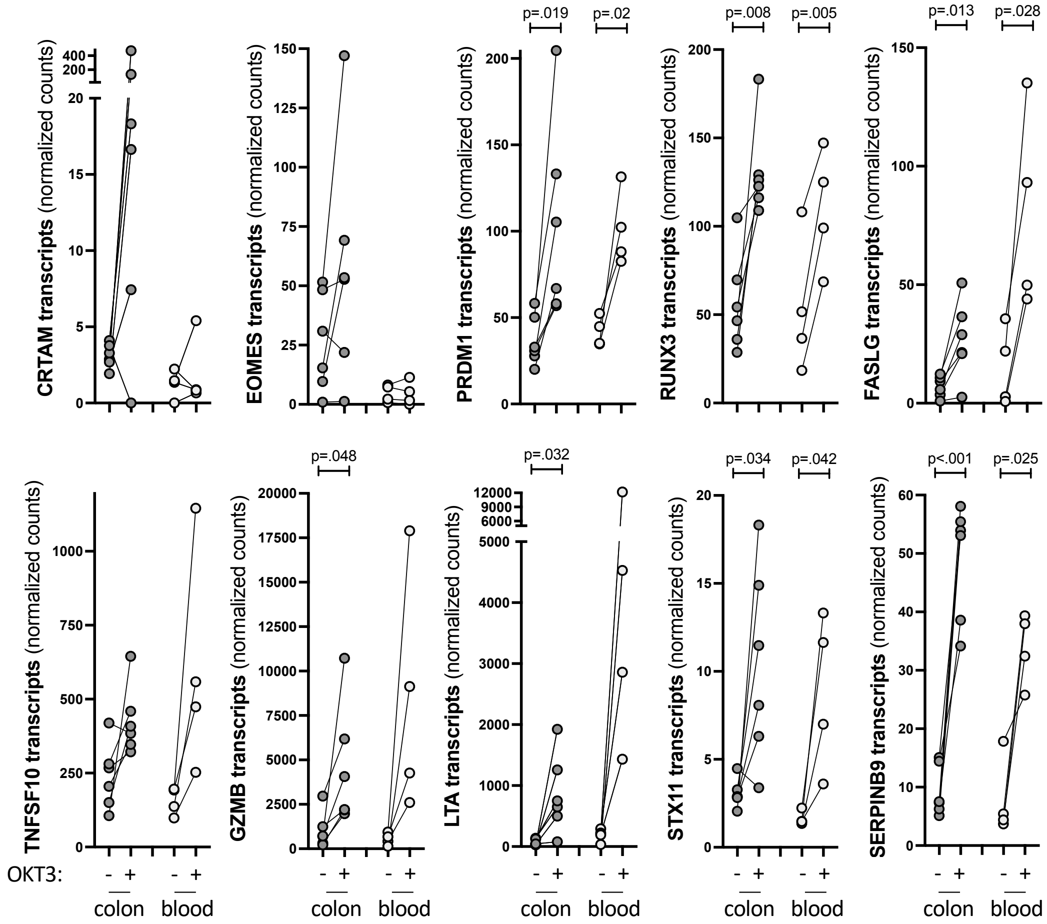

Figure S3. **DP8α Treg clones expressed an array of transcripts involved in cytotoxicity.** Normalized counts for indicated transcripts expressed by individual DP8α Treg clones derived from either the healthy colonic lamina propria or blood are shown. Results are represented for both resting and OKT3-activated clones. Two-sided paired t-test, p<.05 are considered statistically significant.

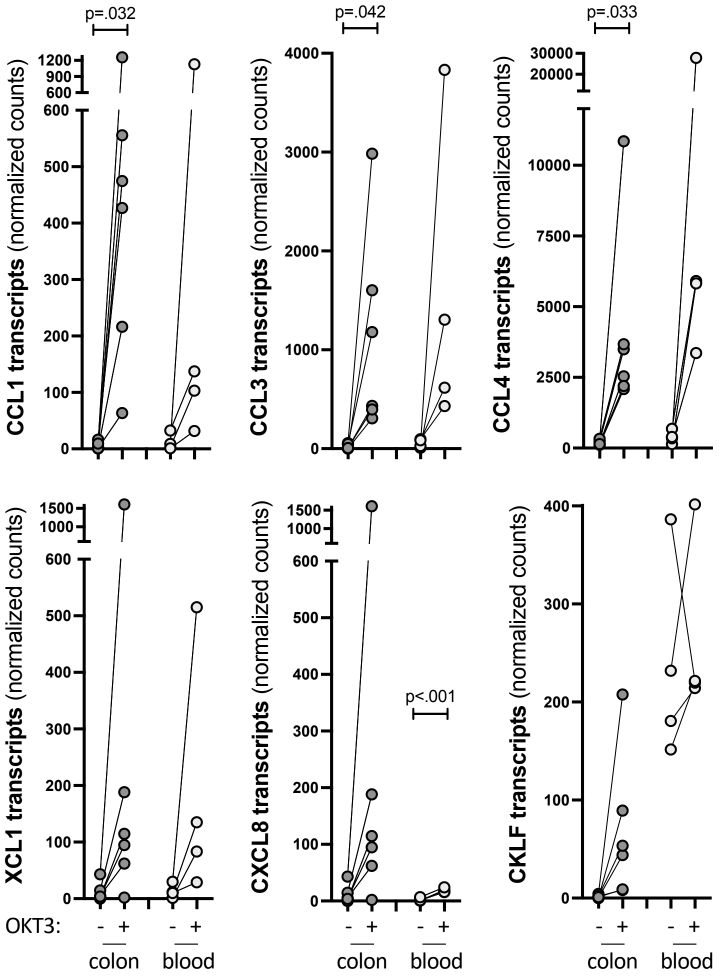

Figure S4. **DP8α Treg clones expressed an array of transcripts involved in chemotaxis.** Normalized counts for indicated transcripts expressed by individual DP8α Treg clones derived from either the healthy colonic lamina propria or blood are shown. Results are represented for both resting and OKT3-activated clones. Two-sided paired t-test, p<.05 are considered statistically significant.

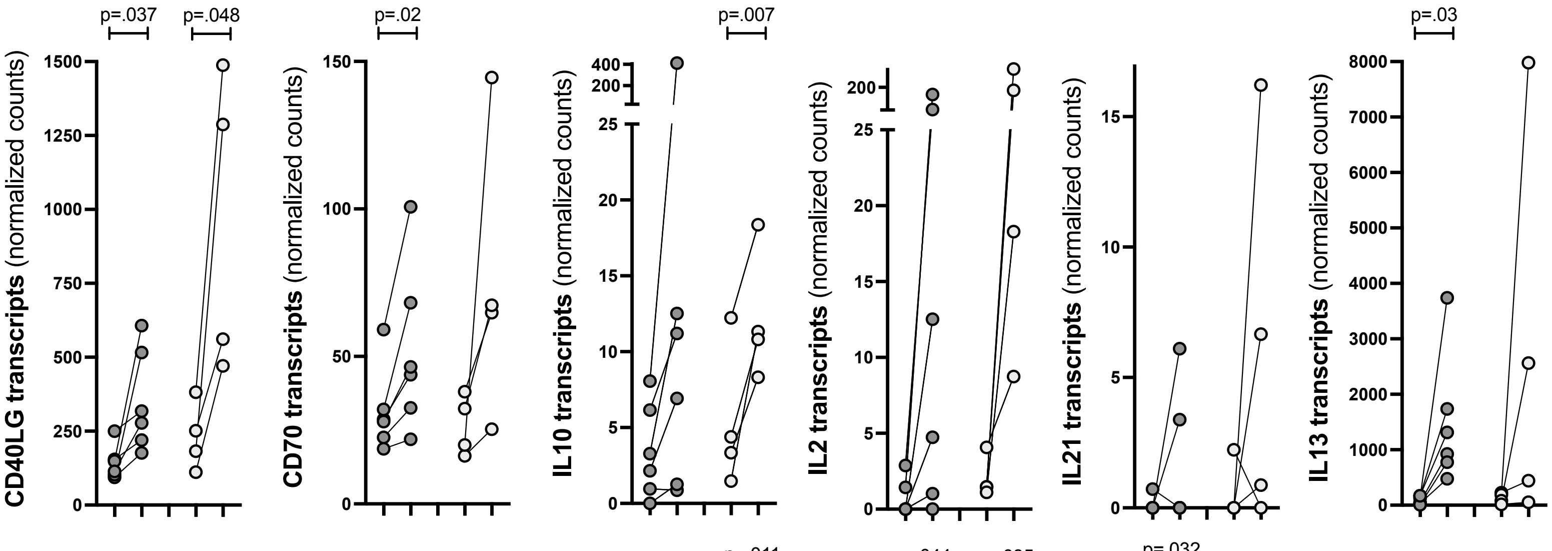

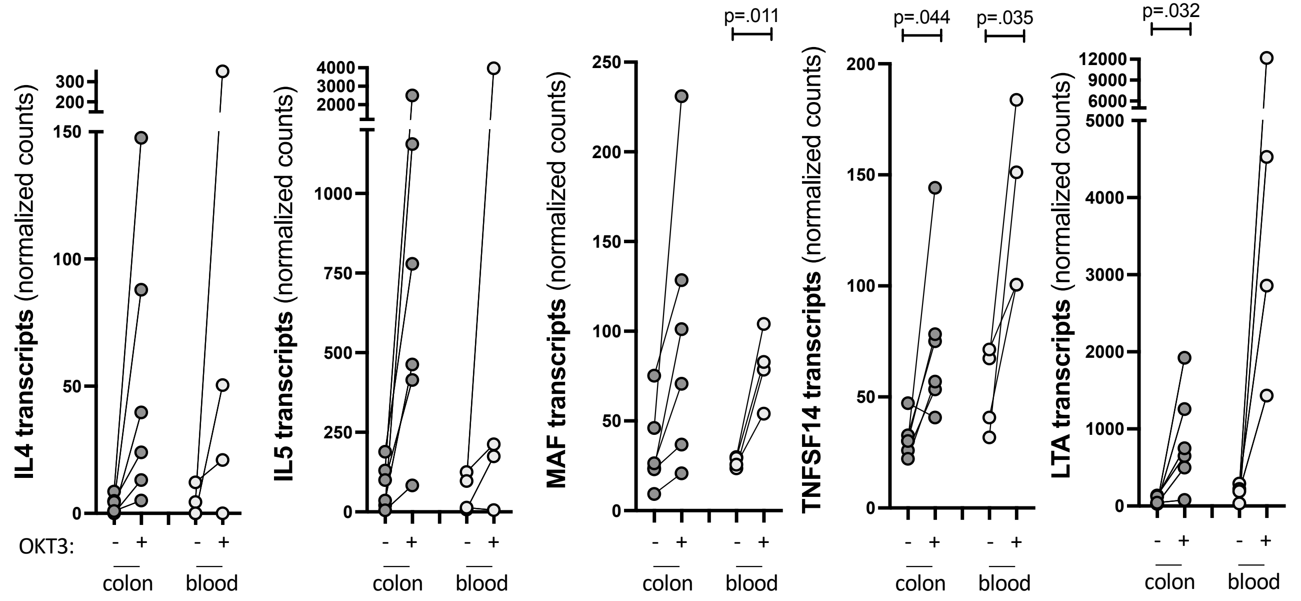

Figure S5. **DP8α Treg clones expressed an array of transcripts involved in B cell help functions.** Normalized counts for indicated transcripts expressed by individual DP8α Treg clones derived from either the healthy colonic lamina propria or blood are shown. Results are represented for both resting and OKT3-activated clones. Two-sided paired t-test, $p<.05$ are considered statistically significant.

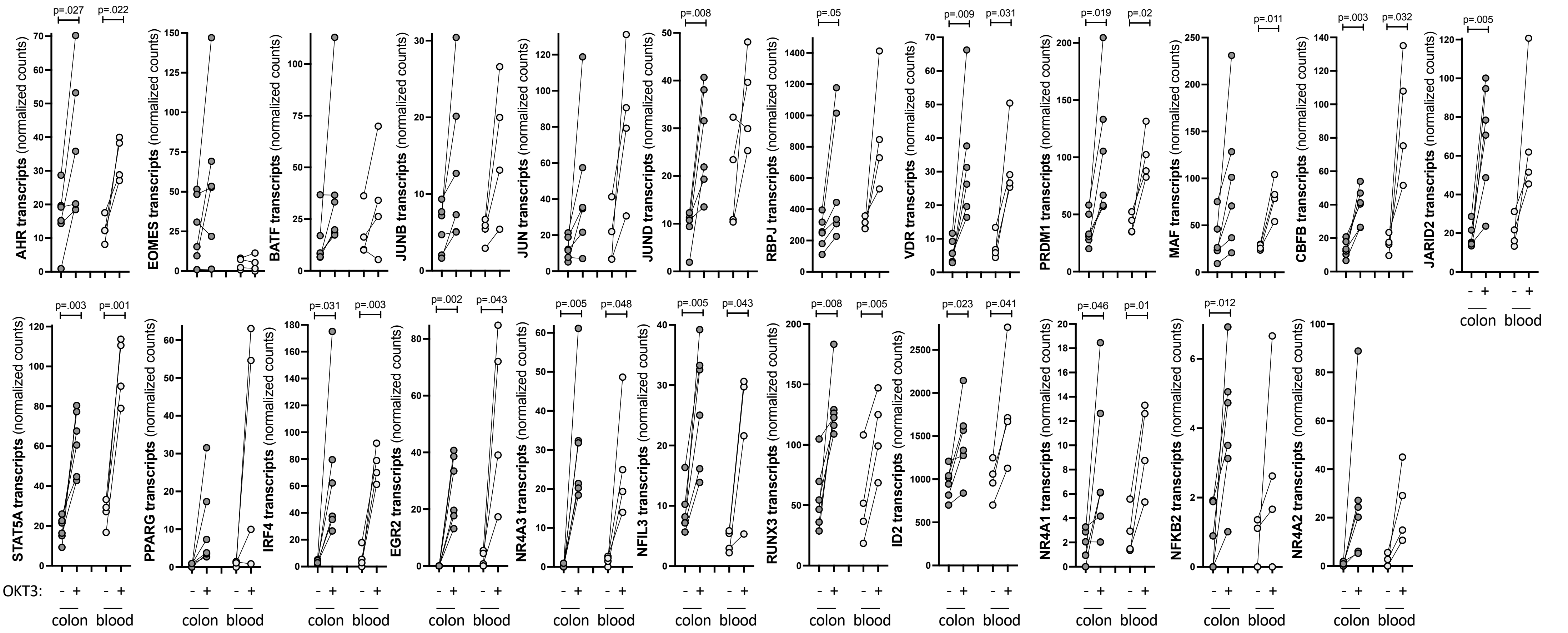

Figure S6. **DP8α Treg clones expressed an array of genes encoding transcription factors.** Normalized counts for indicated transcripts expressed by individual DP8α Treg clones derived from either the healthy colonic lamina propria or blood are shown. Results are represented for both resting and OKT3-activated clones. Two-sided paired t-test, $p<.05$ are considered statistically significant.

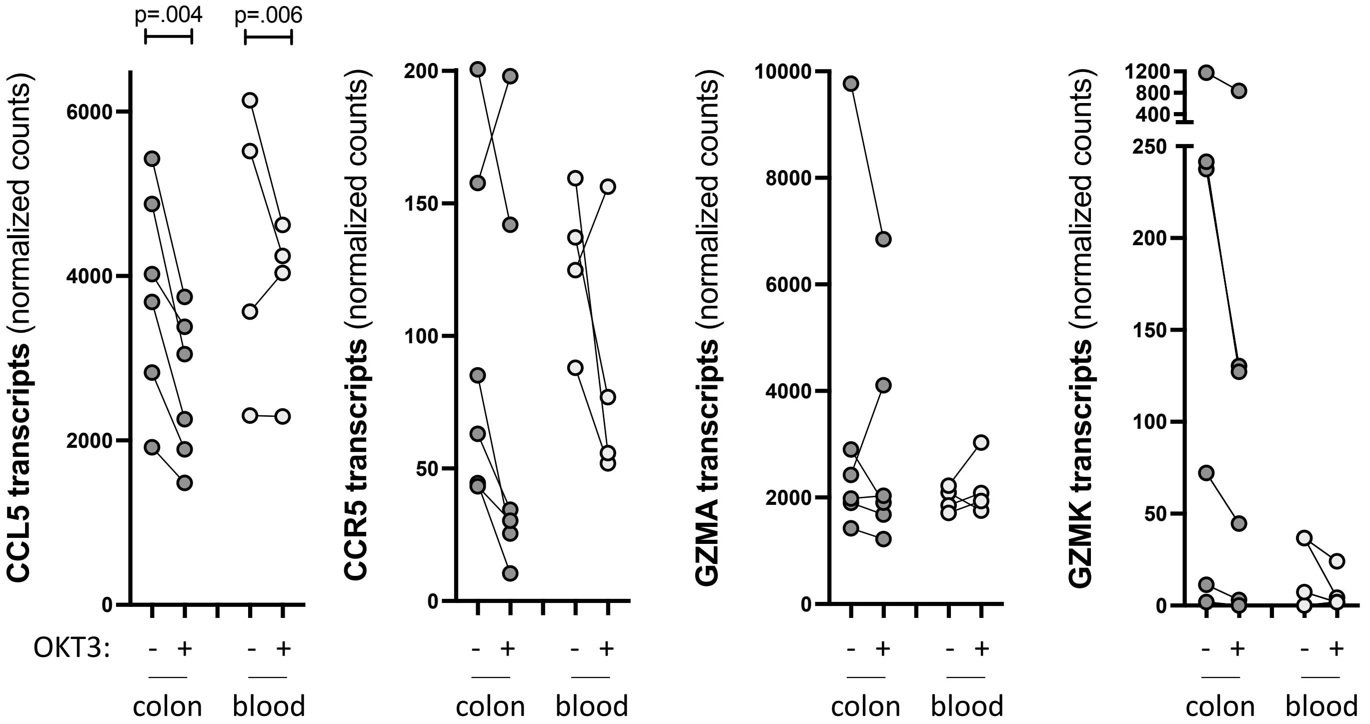

Figure S7. **Several transcripts involved in chemotaxis and lysis functions are expressed by resting DP8α Treg clones and downregulated upon OKT3-activation.** Normalized counts for indicated transcripts expressed by individual DP8α Treg clones derived from either the healthy colonic lamina propria or blood are shown. Results are represented for both resting and OKT3-activated clones. Two-sided paired t-test, $p<.05$ are considered statistically significant.

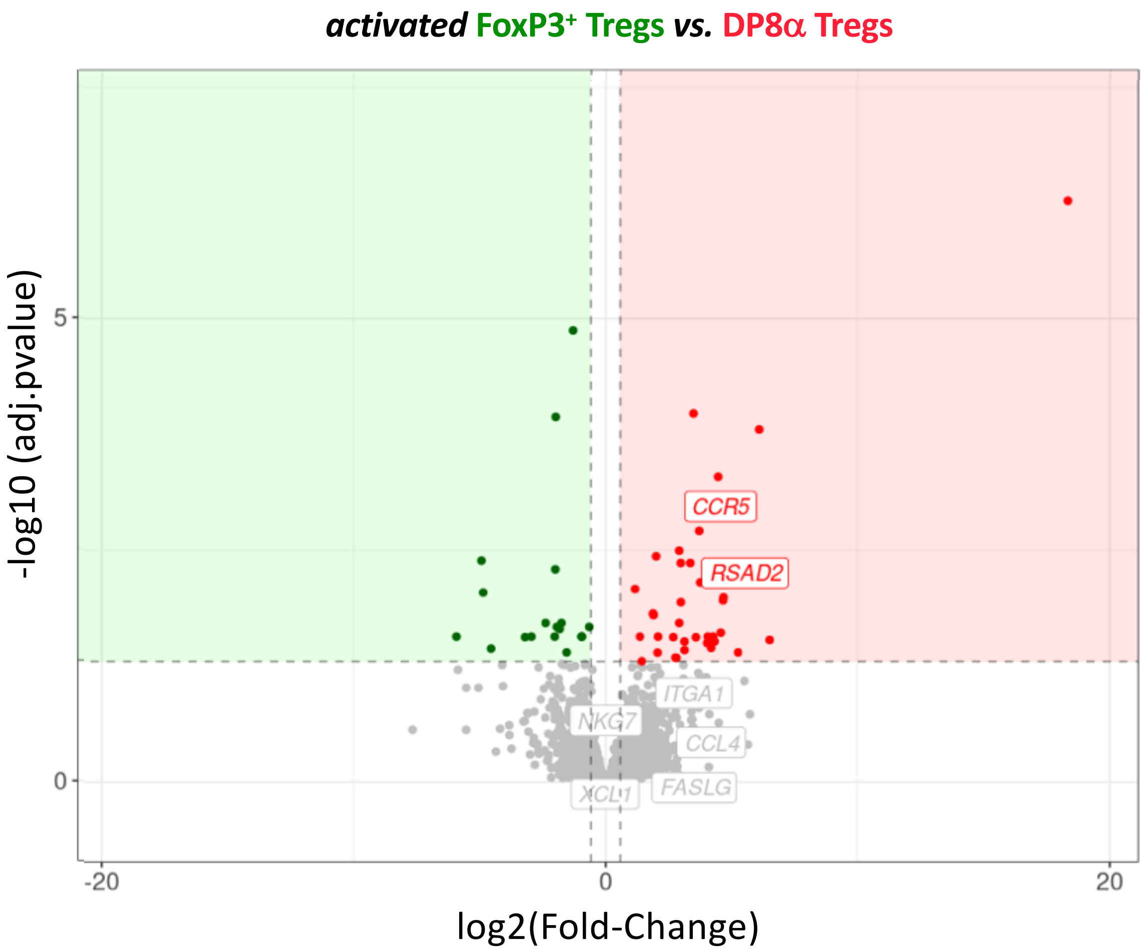

Figure S8. **Differential gene expression between blood-derived OKT3-stimulated DP8α and FoxP3$^+$ Treg clones.** Log 2-fold changes of average expression values between OKT3-stimulated FoxP3$^+$ and DP8α Treg clones are represented as a volcano plot.
